# Type I interferon signaling before hematopoietic stem cell transplantation lowers donor T cell activation via reduced allogenicity of recipient cells

**DOI:** 10.1038/s41598-019-51431-2

**Published:** 2019-10-18

**Authors:** Julius C. Fischer, Michael Bscheider, Sascha Göttert, Erik Thiele Orberg, Stephanie E. Combs, Florian Bassermann, Simon Heidegger, Tobias Haas, Hendrik Poeck

**Affiliations:** 1 Department of Medicine III, Technical University of Munich (TUM), School of Medicine, Klinikum rechts der Isar TUM, Ismaninger Straße 22, 81675 Munich, Germany; 2 Department of Radiation Oncology, Technical University of Munich (TUM), School of Medicine, Klinikum rechts der Isar TUM, Ismaninger Straße 22, 81675 Munich, Germany

**Keywords:** Preclinical research, Bone marrow transplantation

## Abstract

Recent studies highlight immunoregulatory functions of type I interferons (IFN-I) during the pathogenesis of graft-versus-host disease (GVHD) after allogeneic hematopoietic stem cell transplantation (allo-HSCT). We demonstrated that selective activation of IFN-I pathways including RIG-I/MAVS and cGAS/STING prior to allo-HSCT conditioning therapy can ameliorate the course of GVHD. However, direct effects of IFN-Is on immune cells remain ill characterized. We applied RIG-I agonists (3pRNA) to stimulate IFN-I production in murine models of conditioning therapy with total body irradiation (TBI) and GVHD. Using IFN-I receptor-deficient donor T cells and hematopoietic cells, we found that endogenous and RIG-I-induced IFN-Is do not reduce GVHD by acting on these cell types. However, 3pRNA applied before conditioning therapy reduced the ability of CD11c^+^ recipient cells to stimulate proliferation and interferon gamma expression of allogeneic T cells. Consistently, RIG-I activation before TBI reduced the proliferation of transplanted allogeneic T-cells. The reduced allogenicity of CD11c^+^ recipient cells was dependent on IFN-I signaling. Notably, this immunosuppressive function of DCs was restricted to a scenario where tissue damage occurs. Our findings uncover a context (damage by TBI) and IFN-I dependent modulation of T cells by DCs and extend the understanding about the cellular targets of IFN-I during allo-HSCT and GVHD.

## Introduction

Graft-versus-host disease (GVHD) is one of the most frequent and severe complications after allogeneic hematopoietic stem cell transplantation (allo-HSCT). Despite multiple new therapeutic and prophylactic approaches, acute GVHD occurs in as many as 30–50% of allo-HSCT recipients and results in a mortality of 15–30% of all transplanted patients^1,2^. Current treatment options mainly comprise different immunosuppressive drugs that often result in opportunistic infections. Thus, new therapeutic and prophylactic GVHD treatment strategies are urgently needed.

Generally, maintaining epithelial intestinal barrier function is a crucial aspect for the prevention of GVHD. Conditioning therapy-mediated damage to the intestinal barrier can lead to translocation of otherwise luminal gut microbiota, release of danger-associated molecular patterns (DAMPs) and thus activation of recipient antigen presenting cells (APCs) that resisted the conditioning regimen^3^. Strong stimulation of recipient APCs results in enhanced activation, priming and expansion of transplanted allogeneic donor T cells^3^. Following an expansion phase, donor T cells can home to particular organs (gut, liver, skin) where destruction of allogeneic tissue of the recipient ultimately causes morbidity and mortality of GVHD^3^. The complex pathogenesis of GVHD offers various opportunities for therapeutic modulation of donor T cell allo-reactivity.

Several independent studies have recently found that type I interferon (IFN-I) signaling but not type III interferon (IFN-III) activation can modulate GVHD in mice^4–7^. IFN-I receptor deficient (IFNaR1^−/−^) recipient mice developed more severe GVHD, while treatment of allo-HSCT recipients with recombinant IFN-I ameliorated the course of GVHD^5,6^. In this context, innate pattern recognition receptors can be successfully targeted for the potent induction of IFN-I as activation of the RNA receptor system RIG-I/MAVS or the DNA recognition pathway cGAS/STING resulted in prophylactic amelioration of GVHD^5^. Thus, targeting IFN-I-inducing nucleic acid receptor systems is a promising strategy in the development of novel therapies to protect from GVHD^5,8^. Mechanistically, specific activation of RIG-I/MAVS with 5′-triphosphorylated RNA (3pRNA) and cGAS/STING with a 45-base pair non-CpG double-stranded DNA oligonucleotide (interferon stimulatory DNA, ISD^9^), enhanced intestinal epithelial barrier function after allo-HSCT^5^. Hereby, enhanced epithelial regeneration was found to be dependent on IFN-I activation^5^. Importantly, protection of the intestinal barrier function was accompanied by decreased allogeneic T cell activation *in vivo*^5^. However, it remains to be determined how RIG-I/MAVS activation and subsequent IFN-I release modulates allogeneic T cell activation.

Here, we analyzed the effects of RIG-I/MAVS-mediated induction of IFN-I signaling on the priming of allogenic donor T cells after conditioning therapy.

## Results

### Type I interferon receptor signaling in donor T and hematopoietic cells does not contribute to the development of GVHD

**Figure 1:**
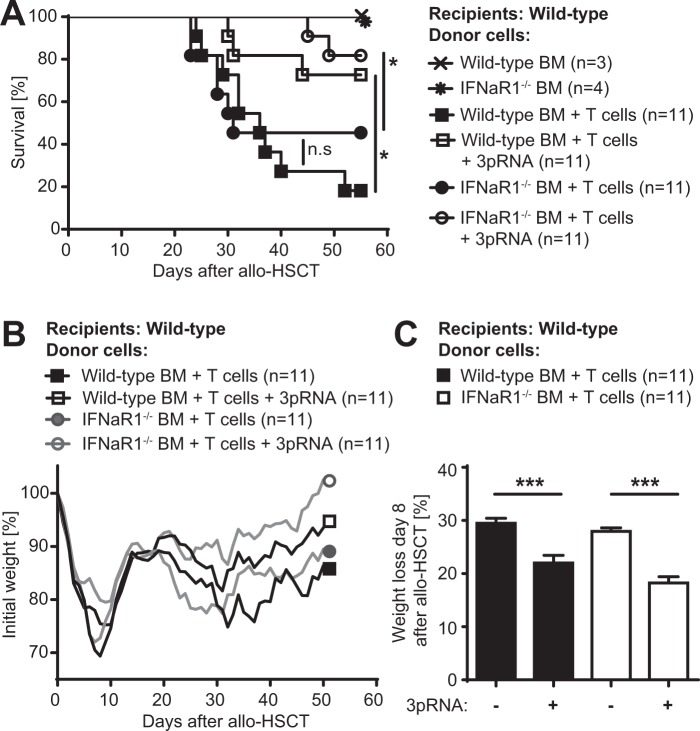
Type I interferon receptor signaling in donor T and hematopoietic cells does not contribute to the development of GVHD. Balb/c mice received conditioning with 9 Gy total body irradiation followed by allogeneic transplantation (C57BL/6 donors) of 5 × 10^6^ bone marrow cells (BM) with or without additional 0.5 × 10^6^ donor T cells. Donor BM cells and T cells were isolated from either *Ifnar1*^+/+^ (WT) or *Ifnar1*^−/−^ mice. (**A)** GVHD-related recipient survival after allo-HSCT. Data are pooled from two independent experiments. (**B**,**C)** Overall recipient weight course after allo-HSCT and weight loss on day 8 after allo-HSCT as a surrogate marker for GVHD severity. Data are pooled from two independent experiments. Animal numbers per group (n) are depicted. Survival was analyzed using the Log-rank test. Weight loss data were analyzed using ordinary one-way ANOVA for multiple comparisons. Significance was set at p values < 0.05, p < 0.01 and p < 0.001 and was then indicated with asterisks (*^,^** and ***). Data are presented as mean ± S.E.M.

First, we addressed the influence of IFN-I on transplanted donor cells. To this end, we used a major mismatch allo-HSCT mouse model and administered allogeneic bone marrow (BM) and T cells derived from either Wild-type (WT) or IFNaR1^−/−^ donor mice. WT recipient mice transplanted with allogeneic BM and T cell grafts derived from IFNaR1^−/−^ donor mice showed similar survival as compared to allo-HSCT recipients transplanted with IFNaR1^+/+^ (WT) BM and T cells (Fig. 1A). Furthermore, lack of IFNaR1 in donor BM and T cell grafts did not influence weight loss of transplanted mice, which is a surrogate marker for the severity of intestinal GVHD (Fig. 1B,C).

Next, we analyzed the contribution of IFNaR1 signaling in donor cells to RIG-I mediated protection against GVHD. We have shown previously that the therapeutic effect of selective RIG-I ligation using 3pRNA in allo-HSCT recipients is mediated via IFN-I signaling^5^. Consistent with this, recipient mice transplanted with wild-type BM and T cells showed reduced weight loss and prolonged survival after injection with the specific RIG-I ligand 3pRNA one day prior to allo-HSCT, as compared to untreated control animals (Fig. 1A,B). Allo-HSCT recipients transplanted with IFNaR1^−/−^ BM and IFNaR1^−/−^ T cells showed a similar beneficial response to 3pRNA treatment with significantly reduced weight loss and mortality (Fig. 1A–C). Taken together, these data demonstrate that neither the development of GVHD nor its amelioration by therapeutic targeting of RIG-I is modulated by IFN-I signaling in hematopoietic donor cells and donor T cells.

### RIG-I activation before conditioning therapy reduces activation of transplanted donor T cells *in vivo*

**Figure 2:**
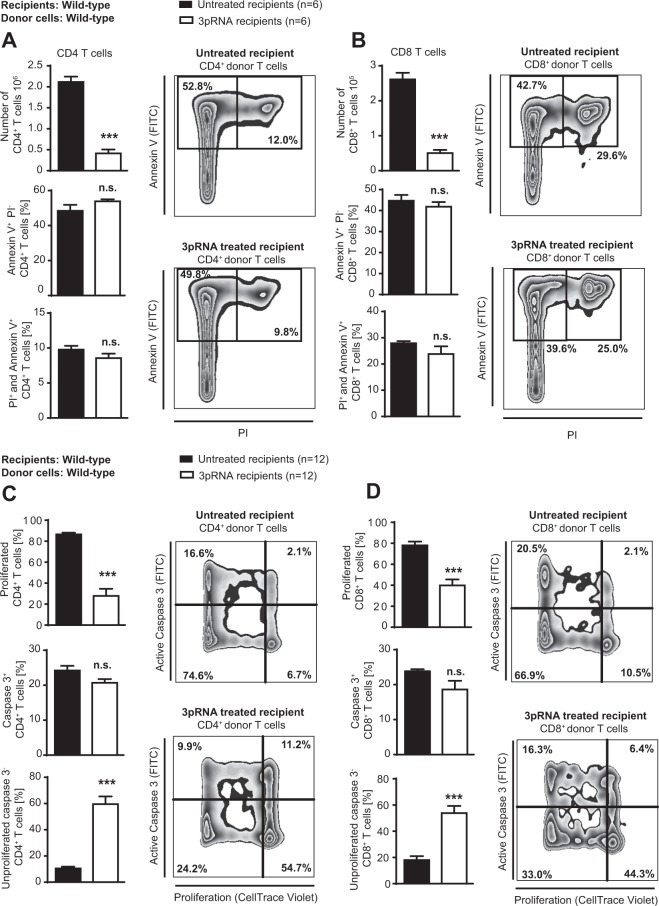
RIG-I activation before conditioning therapy reduces activation of transplanted donor T cells *in vivo*. Balb/c mice received conditioning with 9 Gy total body irradiation followed by allogeneic transplantation of 15 × 10^6^ donor T cells (C57BL/6 donors, h2kb^+^) labeled with CellTrace™ Violet. Splenocytes of recipient mice were counted and analyzed on day 3 after transplantation using flow cytometry. (**A)** Upper panel: Absolute number of donor T cells (CD4^+^ h2kb^+^ splenocytes). Mid panel: Percent Annexin V^+^ PI^−^ CD4^+^ donor T cells. Lower panel: Percent Annexin V^+^ PI^+^ CD4^+^ donor T cells. Representative FACS plots of CD4^+^ h2kb^+^ T cells. Data of one experiment with 6 mice per group. (**B)** Upper panel: Absolute number of donor T cells (CD8^+^ h2kb^+^ splenocytes). Mid panel: Percent Annexin V^+^ PI^−^ CD8^+^ donor T cells. Lower panel: Percent Annexin V^+^ PI^+^ CD8^+^ donor T cells. Representative FACS plots of CD8^+^ h2kb^+^ T cells. Data of one experiment with 6 mice per group. (**C)** Upper panel: Percent proliferated (CellTrace^−^) CD4^+^ donor T cells of all transplanted CD4^+^ donor T cells. Mid panel: Percent active caspase 3^+^ CD4^+^ donor T cells of all transplanted CD4^+^ donor T cells. Percent active caspase 3 negative and non-proliferated CD4^+^ donor T cells (CellTrace^+^) of all transplanted CD4^+^ donor T cells. Representative FACS plots of CD4^+^ h2kb^+^ T cells. Pooled data of two experiments, 12 mice per group. (**D)** Upper panel: Percent proliferated (CellTrace^−^) CD8^+^ donor T cells of all transplanted CD8^+^ donor T cells. Mid panel: Percent active caspase 3^+^ CD8^+^ donor T cells of all transplanted CD8^+^ donor T cells. Percent active caspase 3 negative and non-proliferated CD8^+^ donor T cells (CellTrace^+^) of all transplanted CD8^+^ donor T cells. Representative FACS plots of CD8^+^ h2kb^+^ T cells. Pooled data of two experiments, 12 mice per group. Data were analyzed using two-tailed unpaired t test. Significance was set at p values < 0.05, p < 0.01 and p < 0.001 and was then indicated with asterisks (*^,^** and ***). Data are presented as mean ± S.E.M.

Based on the above findings, we hypothesized that RIG-I activation limits recipient cells to activate transplanted donor T cells after allo-HSCT. Therefore, we analyzed proliferation and cell death of transplanted donor T cells in mice that were either treated with 3pRNA before TBI or left untreated. We found that RIG-I activation with 3pRNA before TBI reduced the absolute number of CD4^+^ and CD8^+^ donor T cells after transplantation, whereas the percentage of Annexin V positive or Propidiumiodid positive T cells was unchanged between 3pRNA treated and untreated recipients (Fig. 2A,B). Next we analyzed the proliferation of transplanted T cells and found that 3pRNA treatment reduced the proliferation of CD4^+^ and CD8^+^ donor T cells (Fig. 2C,D). Finally, we analyzed apoptosis as determined by caspase 3 activation and found that caspase 3 activation of transplanted CD4^+^ and CD8^+^ T cells was comparable in 3pRNA treated and untreated mice. In addition, we found that the percentage of T cells that did not show either proliferation or caspase 3 activation was increased after 3pRNA treatment (Fig. 2C,D). In sum, we found reduced numbers of donor T cells after transplantation, which correlated mechanistically with reduced proliferation but not with enhanced cell death.

### Induction of IFN-I before conditioning therapy with TBI reduces allogenicity of dendritic cells to activate allogeneic T cells

**Figure 3:**
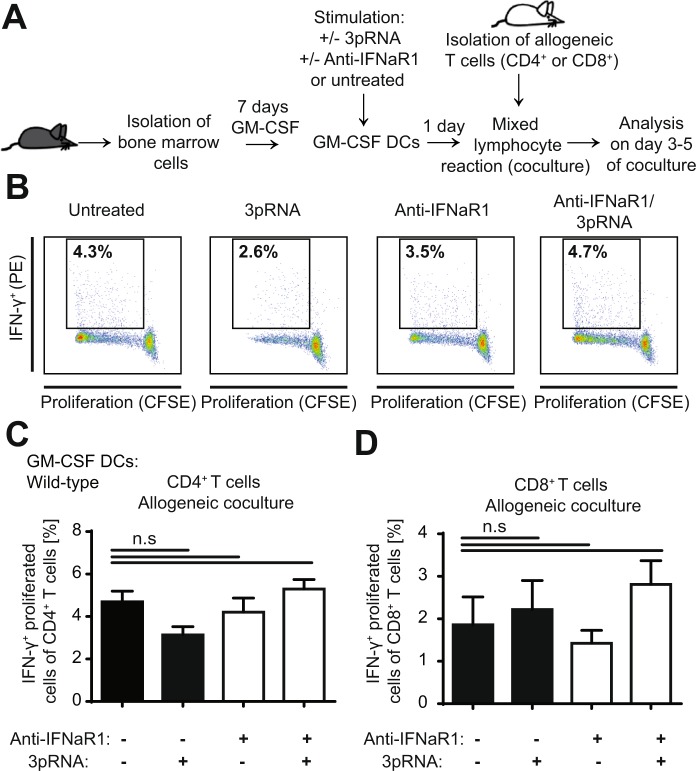
*In vitro* activation of the RIG-I/MAVS/IFN-I pathway in dendritic cells does not significantly influence allogeneic T cell activation. (**A)** Scheme of experimental setup: BM isolated from C57BL/6 WT mice was used to generate BM-derived GM-CSF DCs. GM-SCF DCs were stimulated with 3pRNA with or without additional treatment with anti-IFNaR1. One day later, stimulated DCs were cocultured with allogeneic CD4^+^ or CD8^+^ T cells derived from Balbc/c WT mice. Proliferation and IFN-γ production were analyzed on day 3 (CD8^+^ T cells) or 5 (CD4^+^ T cells) after onset of the mixed lymphocyte reaction (MLR). (**B)** Representative gating strategy of MLR with CD4^+^ T cells: Analysis of live (live/dead stain negative) CD4^+^ lymphocytes. The gate shows the percentage of proliferated (CFSE negative) and IFN-γ^+^ cells of all CD4^+^ T cells on day 5 after onset of the MLR of GM-CSF DCs generated from WT BM. Representative data from one of four experiments. (**C**) Percentage of proliferated and IFN-γ^+^ cells of all CD4^+^ T cells on day 5 after onset of the MLR. Pooled data of four independent experiments. (**D)** Percentage of proliferated and IFN-γ^+^ cells of all CD8^+^ T cells on day 3 after onset of the MLR. Pooled data of four independent experiments. Data were analyzed using two-tailed unpaired t test or ordinary one-way ANOVA for multiple comparisons. Significance was set at p values < 0.05, p < 0.01 and p < 0.001 and was then indicated with asterisks (*^,^** and ***). Data are presented as mean ± S.E.M.

RIG-I-induced IFN-I release may limit recipient antigen presenting cells (APCs) to activate donor T cells resulting in reduced activation of allogeneic T cells. To address this, we first used a conventional mixed lymphocyte reaction (MLR) with *in vitro* generated BM-derived dendritic cells (DCs), which are co-cultured with allogeneic CD4^+^ or CD8^+^ T cells after stimulation with 3pRNA. The advantage of this conventional MLR was to analyze direct RIG-I dependent effects on DC function independent of the pleotropic effects on DCs that may be induced by the conditioning therapy before allo-HSCT. After three to five days of co-culture, we assessed proliferation and IFN-γ production of allogeneic T cells (Fig. 3A). We did not observe significant changes in allogenic T cell activation after DC stimulation with RIG-I-MAVS activating 3pRNA *in vitro* (Fig. 3B–D). Furthermore, blocking of the IFN-I receptor with anti-IFNaR1 antibody did not alter allogeneic CD4^+^ or CD8^+^ T cell activation *in vitro* (Fig. 3B–D).

We therefore postulate that 3pRNA treatment before the conditioning therapy negatively regulates T cell stimulatory responses induced by conditioning. This results in reduced allogeneic T cell activation after allo-HSCT. Therefore, a conventional MLR using BM-derived dendritic cells DCs co-cultured with allogeneic T cells *in vitro* and in the absence of damage cannot mirror this scenario.

**Figure 4:**
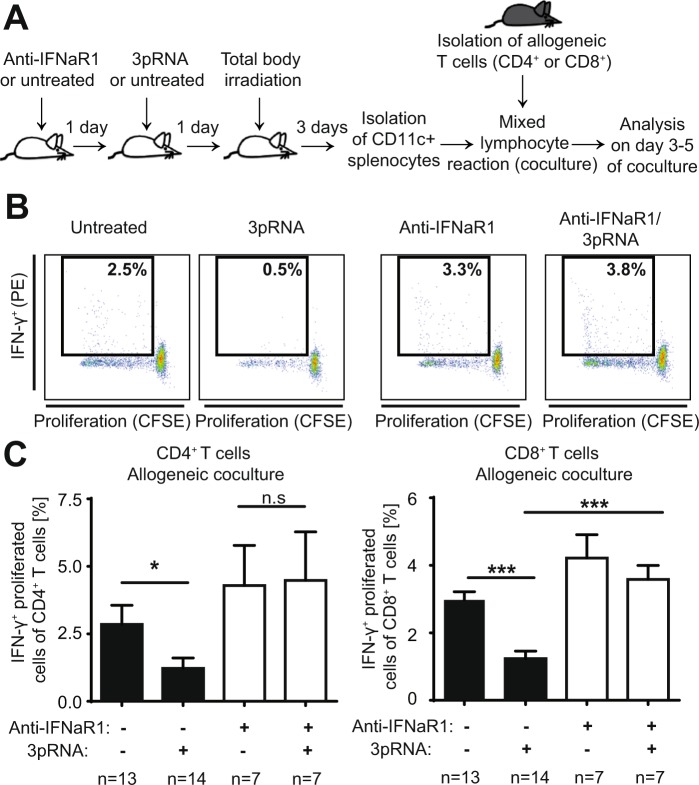
Induction of type I interferon by RIG-I activation prior to conditioning therapy reduces allogenicity of dendritic cells to activate allogeneic T cells. (**A)** Scheme of experimental setup: Balb/c mice were treated with IFNaR1 blocking antibody or were left untreated. One day later mice were treated with 3pRNA or were left untreated. One day later all mice received TBI with 9 Gy. Three days later spleens were harvested and CD11c^+^ cells were isolated and cocultured with allogeneic CD4^+^ or CD8^+^ T cells derived from C57BL/6 mice. Proliferation and IFN-γ production were analyzed on day 3 (CD8^+^ T cells) and 5 (CD4^+^ T cells) after onset of the mixed lymphocyte reaction (MLR). (**B)** FACS gating of T cells after MLR. CD4^+^ T cells were analyzed on day 5 after MLR. Representative data from one of five experiments. **C)** Left panel**:** Percentage of proliferated and IFN-γ^+^ cells of all CD4^+^ T cells on day 5 after onset of the MLR. Right panel: Percentage of proliferated and IFN-γ^+^ cells of all CD8^+^ T cells on day 3 after onset of the MLR. Pooled data of five independent experiments. Animal numbers per group (n) are depicted. Data were analyzed using ordinary one-way ANOVA for multiple comparisons. Significance was set at p values < 0.05, p < 0.01 and p < 0.001 and was then indicated with asterisks (*^,^** and ***). Data are presented as mean ± S.E.M.

We therefore aimed to analyze the allogenicity of recipient DCs after *in vivo* 3pRNA treatment and *in vivo* conditioning therapy. On day 3 after allo-HSCT, high amounts of transplanted donor T cells are located within the spleen of recipient mice, before they begin to infiltrate GVHD effector organs such as the intestine^10^. We thus aimed to analyze the potency of splenic recipient CD11c^+^ DCs to activate allogeneic T cells after conditioning therapy. Consequently, we used an *ex vivo* MLR to mimic the interaction of transplanted donor T cells with recipient DCs after conditioning therapy and allo-HSCT in the host. We isolated splenic CD11c^+^ DCs on day 3 after TBI from mice that had already been treated with 3pRNA prior to irradiation (Fig. 4A). We then subjected isolated CD11c^+^ cells to co-culture with CD4^+^ or CD8^+^ T cells isolated from allogeneic mice. After three to five days of co-culture, we assessed DC allogenicity by measuring proliferation and IFN-γ production of T cells (Fig. 4A,B). DCs isolated from irradiated mice activated allogenic CD4^+^ and CD8^+^ T cells (Fig. 4B,C). Pretreatment of DC donor mice with 3pRNA prior to radiation therapy and cell harvest significantly decreased proliferation and IFN-γ production of subsequently co-cultured allogenic T cells (Fig. 4B,C). To analyze the role of IFNaR1 activation in our model, we additionally pretreated recipients with IFNaR1-blocking antibody on day -2 and before injection of 3pRNA (day -1) (Fig. 4A). Pre-treatment with IFNaR1-blocking antibody abrogated the ability of 3pRNA to limit allogeneic T cell activation in our *ex vivo* MLR (Fig. 4B,C). We conclude that *in vivo* pretreatment of mice with 3pRNA before conditioning therapy lowers the allogenicity of splenic DCs via an IFN-I dependent mechanism.

### Tissue damage induced by irradiation is a pre-requisite for RIG-I induced immunosuppression

**Figure 5:**
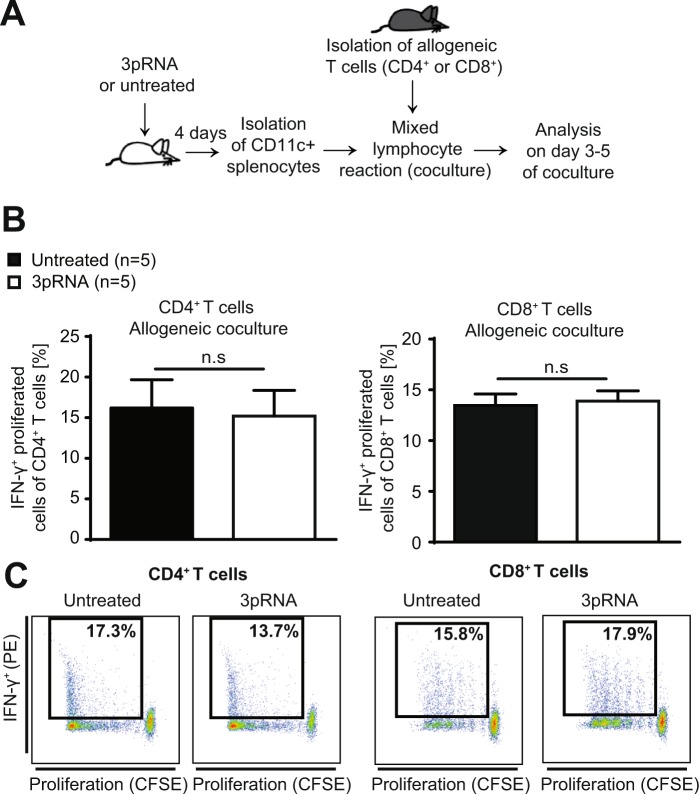
Tissue damage induced by irradiation is a pre-requisite for RIG-I induced immunosuppression. (**A)** Scheme of experimental setup: Balb/c mice were treated with 3pRNA or were left untreated. Four days later, splenic CD11c^+^ cells were isolated and cocultured with allogeneic CD4^+^ or CD8^+^ T cells derived from C57BL/6 mice. Proliferation and IFN-γ production were analyzed on day 3 (CD8^+^ T cells) or 5 (CD4^+^ T cells) after onset of the mixed lymphocyte reaction (MLR). (**B)** Percentage of proliferated and IFN-γ^+^ cells of all CD4^+^ T cells on day 5 after onset of the MLR (left panel). Percentage of proliferated and IFN-γ^+^ cells of all CD8^+^ T cells on day 3 after onset of the MLR (right panel). Pooled data of two independent experiments. (**C)** FACS gating of data presented in B. FACS plots of CD4^+^ T cells (left panel) and CD8^+^ T cells (right panel). Representative data from one of two experiments. Donor animal numbers per group (n) are depicted. Data were analyzed using two-tailed unpaired t test. Significance was set at p values < 0.05, p < 0.01 and p < 0.001 and was then indicated with asterisks (*^,^** and ***). Data are presented as mean ± S.E.M.

Next, we examined whether 3pRNA-induced RIG-I signaling directly influenced the allogenicity of dendritic cells in the absence of tissue damage induced by pre-transplant conditioning such as radiation therapy. Therefore, we isolated splenic CD11c^+^ DCs from mice that had been treated with 3pRNA but did not undergo subsequent irradiation (Fig. 5A). We found that 3pRNA treatment of naive mice did not influence the potency of isolated DCs to activate allogeneic CD4^+^ or CD8^+^ T cells (Fig. 5B,C). We thus conclude that 3pRNA treatment and subsequent IFN-I release in mice *per se* do not directly influence the allogenicity of DCs. Rather, 3pRNA pre-treatment of mice seems to dampen the immune activating effects of tissue damage on APCs to stimulate allogeneic T cells.

## Discussion

Recent studies highlighted the potential of IFN-I to modulate intestinal epithelial barrier damage and regeneration^5,11–13^. Furthermore, several independent groups have shown that IFN-I activation can ameliorate GVHD after allo-HSCT^4–6^. We reported previously that treatment of recipient mice with IFN-I inducing targeted therapy of RIG-I prior to allo-HSCT results in reduced development of GVHD. Mechanistically, IFN-I activation promoted intestinal barrier function and epithelial regeneration after conditioning therapy and during GVHD. We could link this enhanced mucosal integrity to reduced proliferation and IFN-γ production of transplanted T cells *in vivo*^5^. We now characterized the details of the influence of IFN-I signaling on donor T cell activation after allo-HSCT. We found that i) IFN-I signaling in donor hematopoietic and T cells does not influence GVHD after allo-HSCT and ii) targeted therapy of RIG-I ameliorates GVHD independent of IFN-I signaling in donor hematopoietic and T cells. Furthermore, RIG-I activation before allo-HSCT resulted not only in reduced proliferation as previously described^5^ but also reduced apoptosis and a large fraction of inactive transplanted donor T cells. Mechanistically, we found that *in vivo* 3pRNA treatment reduced the potency of isolated CD11c^+^ recipient DCs to activate allogeneic donor T cells in an IFN-I dependent manner. Importantly, this immunosuppressive role of IFN-I signaling seems to be restricted to a scenario of genotoxic tissue damage as neither RIG-I activation and IFN-I induction in naive (unirradiated) mice altered allogeneic T cell activation. Consistent with this, *in vitro* activation of the RIG-I/MAVS/IFN-I pathway in *in vitro* generated DCs failed to alter the activation of *allogeneic* CD4^+^ or CD8^+^ T cells during conventional MLRs. Our data are contradictory to the well-established role of RIG-I induced IFN-Is in mediation enhanced cross-priming of *syngeneic* CD8^+^ T cells^14,15^. However, little is currently known about context dependent roles of IFN-Is on APC function and their modulation of T cell response. Therefore, further mechanistic studies are needed to determine how genotoxic tissue damage such as irradiation modulates the effects of I RIG-I/cGAS induced IFN-I on allogeneic and syngeneic T cell cross priming.

**Figure 6:**
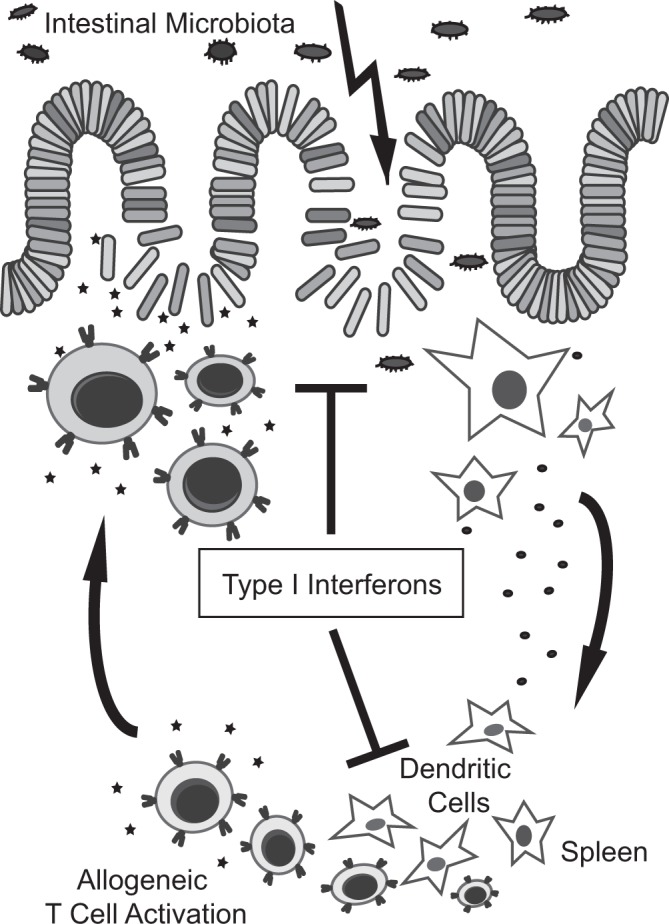
Type I Interferon modulates both, intestinal barrier function after allo-HSCT and allogeneic T cell priming by recipient APCs. The scheme shows two effects of IFN-I activation during conditioning therapy and allo-HSCT. (I) IFN-I reduces intestinal epithelial cell damage and protects intestinal barrier function during allo-HSCT and GVHD. (II) IFN-I limits the allogenicity of CD11c^+^ recipient APCs to prime allogeneic donor T cells.

We have previously published that allo-HSCT recipient mice with a selective deficiency for IFN-I receptor signaling in CD11c^+^ dendritic cells (CD11cre^pos^IFNaR1^floxed^ mice) still develop reduced GVHD after treatment with 3pRNA prior to allo-HSCT^5^. Putting our new data and our previously published results in context, we conclude that 3pRNA-induced IFN-I does not directly target CD11c^+^ DCs to reduce their allogenicity to prime donor T cells but acts via an indirect mechanism. One explanation could be that IFN-I protects the intestinal barrier function after allo-HSCT resulting in reduced APC and donor T cell activation and intestinal GVHD (Fig. 6)^5^. This conclusion is in line with a recent study showing that metabolites derived from the intestinal microbiota act via modulation of IFN-I signaling to limit damage to the intestinal epithelium during GVHD^4^. In both cases, protection of mucosal integrity results in reduced systemic inflammation and reduced systemic DC activation after allo-HSCT^3,16^. Alternatively, IFN-I may act on distinct target cells such as stromal cells and indirectly modulate the initial activation of recipient DCs and their interaction with transplanted donor T cells after allo-HSCT. Such reduced activation of recipient DCs ultimately results in reduced priming of transplanted donor T cells (Fig. 6). In sum, our findings improve our knowledge about the pleotropic effects of IFN-I signaling during allo-HSCT and GVHD and may be relevant for the development of new approaches to modulate donor T cell activation after allo-HSCT.

## Material and Methods

### Mice and animal studies

Animal studies were approved by the local regulatory authority (Regierung von Oberbayern, Munich). All experiments were performed in accordance with the relevant guidelines and regulations. C57BL/6 (H-2kb) and Balb/c (H-2kd) were purchased from Janvier Labs (France). IFNaR1-deficient mice (*Ifnar1*^−/−^) were provided by Ulrich Kalinke and were previously described^17^. Mice were between 6 and 10 weeks of age at the onset of experiments.

### Induction of GVHD after allo-HSCT with myeloablative TBI

Induction of GVHD after allo-HSCT with myeloablative TBI using a major mismatch (H-2kd/H-2kb) GVHD mouse model were performed as previously described^18^. Briefly, Balb/c recipients were injected with 5 × 10^6^ BM cells directly after myeloablative TBI with 2 × 4.5 Gy. Radiation was performed using the Gulmay RS225A irradiation device (Gulmay Medical, Camberley, UK) at a dose rate of 0.95 Gy/min (15 mA, 200 keV). Some mice additionally received 0.5 × 10^6^ C57BL/6 purified donor T cells. Donor T cells and bone marrow cells were isolated from either WT mice or IFNaR1-deficient mice as indicated in the figure legends. Weight loss was monitored daily after allo-HSCT.

### Mixed lymphocyte reaction with bone marrow derived GMCSF dendritic cells

Mixed lymphocyte reaction (MLR) were performed as described previously^19^. In brief, BM cells were harvested from murine femur and tibia of C57BL/6 mice and erythrocytes were lysed with red blood cell lysis buffer. BMDCs were generated by culturing BM cells in complete RPMI medium (PMI‐1640 medium (Invitrogen) supplemented with 10% FCS, 3 mM l‐glutamine, 100 U/mL of penicillin, and 100 μg/mL of streptomycin) supplemented with 20 ng/mL GM‐CSF. Fresh medium was added on the 3rd and 6th day after the start of the culture. GMCSF-DCs were harvested on day 7 using PBS supplemented with 2 mM EDTA. 25 × 10^3^ GMSCF-DCs (C57BL/6) were plated in 96-U-well plates. DCs were treated with 3pRNA if indicated. To do so, DCs were transfected with 3pRNA (1 µg/ml) complexed in Lipofectamin 2000 (Life Technologies, Darmstadt, Germany) according to the manufacturer’s protocol. In indicated experiments GMCSF-DCs were treated with anti-IFNaR1 blocking antibody (10 ug/ml) (Clone: MAR1-5A3, BioXCell, West Lebanon, NH). On the next day, 10^5^ CD4^+^ or CD8^+^ T cells extracted from the spleens of naïve WT mice (Bab/c) were added to the GMSCF-DCs (C57BL/6). Proliferation of T cells was analyzed using the CellTrace™ CFSE Cell Proliferation Kit (Thermo Fisher Scientific). CD4^+^ or CD8^+^ T cells labeled with CFSE were analyzed on day 3 (CD8^+^ T cells) or day 5 (CD4^+^ T cells) after coculture using flow cytometry.

### Mixed lymphocyte reaction with isolated CD11c^+^ splenocytes

Spleens were cut into small pieces and incubated with PBS^+Ca/+Mg^ supplemented with FCS (10%) and Collagenase II (200 U/ml; Worthington) at 37 °C for 45 min. Remaining tissue was mashed slightly and filtered through a 100 μm strainer (BD 352360). Afterwards, CD11c^+^ cells were purified using CD11c^+^ MACS positive selection. CD11c^+^ cells were washed, resuspended in complete media and 1 × 10^4^ cells were cocultured with 1 × 10^5^ allogeneic T cells purified from splenocytes derived from C57BL/6 mice (CD4 or CD8 MACS positive selection). In indicated experiments, Balb/c mice were exposed to TBI with 9 Gy three days prior to isolation of CD11^+^ splenocytes. Proliferation of T cells was analyzed using the CellTrace™ CFSE Cell Proliferation Kit (Thermo Fisher Scientific). CD4^+^ or CD8^+^ T cells labeled with CFSE were analyzed on day 3 (CD8^+^ T cells) or day 5 (CD4^+^ T cells) after coculture using flow cytometry.

### Flow cytometry

All antibodies were purchased from BD Bioscience or BioLegend. We used LIVE/DEAD Fixable Dead Cell Stains and the Dead Cell Apoptosis Kit with Annexin V FITC and Propidium Iodide (PI, Thermo Fisher Scientific) according to the manufacturer’s instructions. For intracellular cytokine staining, T cells were activated with 80 nM Phorbol-12-myristat-13- acetat (PMA; Sigma), 1 μM ionomycin (Merck Millipor) and Brefeldin A for 4 hours. The Foxp3 Transcription Factor Fixation/Permeabilization Kit (eBioscience) was used according to manufacturer’s instructions. Data acquisition and analysis was performed using a FACSCanto II (BD Bioscience) and Flow Jo software (Tree Star).

### T cell analysis *in vivo*

*In vivo* T cell proliferation was performed as previously described^5^. T cell preparation was performed as described above. T cells were stained with CellTrace™ Violet Cell Proliferation Kit (Thermo Fisher Scientific) according to the manufacturer’s instructions. 12 minutes after staining at 37 °C, T cells were washed and counted. 15 × 10^6^ stained cells were transplanted into lethally irradiated allogeneic recipients as described above. Spleens of allo-HSCT recipients were harvested on day 3 and analyzed with FACS. Caspase 3 activation was analyzed as previously described^20^. Active caspase-3 stain was performed directly before further viability and cell surface staining using the CaspGLOW Fluorescein Active Caspase-3 Staining Kit (Thermo Fisher Scientific) according to the manufacturer’s instructions. Whole splenocytes were incubated with the staining reagent for 60 minutes at 37 °C. Afterwards, surface staining was performed as described above.

### Reagents

Double-stranded *in vitro*-transcribed 3pRNA (sense, 5′-UCA AAC AGU CCU CGC AUG CCU AUA GUG AGU CG-3′) was generated as described^21^.

### Drug treatment

Drug treatments were performed as previously described^5^. Mice were treated on indicated time points with 25 µg 3pRNA. 3pRNA was complexed in 3.5 µl *in vivo*-jetPEI (Polyplus) and was injected intravenously. In some experiments, mice were injected intraperitoneally with 500 µg IFNaR1 blocking antibody (Clone: MAR1-5A3, BioXCell, West Lebanon, NH).

### Statistics

Animal numbers per group (n) are depicted in the figure legends. GraphPad Prism version 6 was used for statistical analysis. Survival was analyzed using the Log-rank test. Differences between means of experimental groups were analyzed using two-tailed unpaired t test or ordinary one-way ANOVA correspondingly to the distribution shape of our observations. We used ordinary one-way ANOVA for multiple comparisons and always performed Dunnett’s test for Multiple-test corrections. Applied statistical tests are indicated in the figure legends. Significance was set at p values < 0.05, p < 0.01 and p < 0.001 as indicated with asterisks (*^,^** and ***, respectively). Data are presented as mean ± S.E.M.

## Data Availability

Data generated in the present study are available from the corresponding author upon reasonable request.
